# A Natural Product Telomerase Activator Lengthens Telomeres in Humans: A Randomized, Double Blind, and Placebo Controlled Study

**DOI:** 10.1089/rej.2015.1793

**Published:** 2016-12-01

**Authors:** Laura Salvador, Gunasekaran Singaravelu, Calvin B. Harley, Peter Flom, Anitha Suram, Joseph M. Raffaele

**Affiliations:** ^1^ Anti-aging Group Barcelona, Barcelona, Spain.; ^2^ T.A. Sciences, Inc., New York, New York.; ^3^ Independent Consultant, Murphys, California.; ^4^ Peter Flom Consulting, New York, New York.; ^5^ PhysioAge Systems, LLC, New York, New York.

**Keywords:** telomere length, TA-65, *Astragalus*, telomerase, randomized, placebo controlled trial

## Abstract

TA-65 is a dietary supplement based on an improved formulation of a small molecule telomerase activator that was discovered in a systematic screening of natural product extracts from traditional Chinese medicines. This study summarizes the findings on telomere length (TL) changes from a randomized, double blind, placebo controlled study of TA-65 over a 1 year period. The study was conducted on 117 relatively healthy cytomegalovirus-positive subjects aged 53–87 years old. Subjects taking the low dose of TA-65 (250 U) significantly increased TL over the 12 months period (530 ± 180 bp; *p* = 0.005), whereas subjects in the placebo group significantly lost TL (290 ± 100 bp; *p* = 0.01). The high dose of TA-65 (1000 U) showed a trend of improvements in TL compared with that of the placebo group; however, the improvements did not reach statistical significance. TL changes in the low-dose group were similar for both median and 20th percentile TLs. The findings suggest that TA-65 can lengthen telomeres in a statistically and possibly clinically significant manner.

## Introduction

TA-65 was discovered as a chemically defined small molecule activator of telomerase in the year 2000 from an empirical screen of natural product extracts from traditional Chinese medicines.^1,2^ (Patent number: US7846904). Since that time, there have been research and observational studies on TA-65 in humans and animal models supporting improvements in biomarkers of aging, including immune, cardiovascular, metabolic, bone, and inflammatory markers, without significant signs of toxicity.^2–4^

The formulation (TA-65MD) is manufactured under the regulations of current good manufacturing practice (cGMP); it is designated as GRAS (generally recognized as safe) for use in a medical food and is sold as a dietary supplement by the company TA Sciences.

Interest in TA-65 as a telomerase activator is largely driven by the potential health benefit of telomere maintenance. Without telomerase, telomeres gradually shorten with each cell division due to the “end replication problem,” oxidative stress, and other natural DNA processing at chromosome ends, ultimately triggering cell senescence, that is, the loss of cell replication capacity and ensuing tissue degeneration when telomeres become critically short.^5^ There is abundant evidence that telomerase activation can help maintain and/or lengthen telomeres^6–8^ and in some cases restore tissue and organ function that has been compromised by critical telomere shortening.^9^ However, to date, there have been no blinded, placebo controlled human studies of TA-65. This report provides the first evidence from a randomized, double blind, placebo controlled study that dietary supplementation with TA-65 has the ability to lengthen telomeres and potentially improve health outcomes in humans, with no observed safety concerns.

Cytomegalovirus (CMV) infects the majority of the population worldwide asymptomatically. Seventy to eighty percent of individuals by the age of 50 are infected with CMV. CMV has been implicated in decreased T-cell immunity, associated immunosenescence, and decrease in the T-cell receptor repertoire, causing clonal expansion of senescent CD8^+^CD28^−^ T cells with a proinflammatory profile.^10^

Recent studies also suggest that CMV infections are associated with increased mortality in the elderly and are a potential factor in the development of cardiovascular disease among immuno-compromised individuals.^11,12^ Here we investigated whether TA-65 can alleviate telomere attrition in CMV^+^ subjects, to support our previous observational study finding that TA-65 appears to preferentially lengthen critically short telomeres in CMV^+^ subjects.^1^ This study is aimed at understanding telomere length (TL) changes in CMV^+^ subjects taking the telomerase activator TA-65 in comparison with the placebo group.

## Materials and Methods

### Study design

This is a randomized, double blind, placebo controlled, parallel group study with three arms. Subjects were randomized to placebo, low-dose, or high-dose groups using a random number table. The principle investigator (PI) and subjects were blinded until the completion of the study. After initial screening (168 subjects), a total of 117 subjects were recruited and 97 subjects completed the study. Forty-five subjects received TA-65: 23 subjects received one TA-65 capsule (250 U) and three placebo capsules; 22 subjects received four TA-65 capsules, each consisting of 250 U of TA-65 (*i.e.*, 1000 U/4 capsules). Fifty-two subjects received four placebo capsules. The study involved 104-day cycles consisting of 90 days of taking product or placebo, followed by 14 days of abstinence from taking the test materials. The trial was run for a period of 1 year. The subjects had six visits during the study: preselection, day 0 (baseline), at 3, 6, 9, and 12 months (final visit). The capsules were taken on an empty stomach in the morning. After baseline testing, subjects were given 3 months' supply of the pills, which they took home for consumption. After baseline, additional visits to the clinic were scheduled each 3 months until the end of the study. The PI, Dr. Salvador checked to see that all the pills given at the prior visit had been consumed to confirm compliance.

The study was conducted in Barcelona, Spain. All the subjects were from Barcelona except one, who was from Malaga (South of Spain). Inclusion criteria were subjects with IgG antibodies positive for CMV, aged between 53 and 87 years old, and who were able to sign informed consent. Exclusion criteria were subjects with active carcinoma, a prior history of cancer, severe infectious diseases (Hepatitis C, Hepatitis V, HIV, and syphilis), autoimmune diseases, hormonal therapy, prior intake of TA-65, or nutritional supplements enriched with Omega-3. The male to female ratio was 1.25.

### Blood collection

Blood was collected five times during the study: at day 0, at 3, 6, 9, and 12 months. Blood was tested for the clinical biomarkers, and an aliquot was used to isolate peripheral blood mononuclear cells (PBMCs) for the high-throughput measurement of TL by fluorescent *in situ* hybridization (FISH).

### Measurement of TL

Median TL in PBMCs was measured by Life Length (Spain) using the high throughput (HT) quantitative fluorescence *in situ* hybridization (Q-FISH) technique. This method is based on a Q-FISH method modified for cells in interphase.^13^ In brief, telomeres are hybridized with a fluorescent peptide nucleic acid (PNA) probe that binds to telomeric repeats (sequence: Alexa488-OO-CCCTAACCCTAACCCTAA, Panagene). Images of nuclei and telomeres are captured by a high-content screen system. The intensity of the fluorescent signal from telomeric PNA probes that hybridize to a given telomere is linearly proportional to the length of the telomere. Intensities of fluorescence are translated to TLs by comparing the obtained intensities of fluorescence versus a standard regression curve built with control cell lines of known TL.

On the processing day, samples and control cell lines were thawed at 37°C and cell counts and viability were determined. Cells were seeded in clear bottom black-walled 384 well plates at a fixed density with five replicates of each PBMC sample and eight replicates of each control cell line. Cells were fixed with methanol/acetic acid (3/1, vol/vol). After hybridization *in situ* with the PNA probe, cells were washed and DAPI added for DNA staining. Quantitative image acquisition and analysis were performed on a High Content Screening Opera System (Perkin Elmer) using the Acapella software, Version 1.8 (Perkin Elmer). Images were captured using a 40 × 0.95 NA water immersion objective. UV and 488 nm excitation wavelengths were used to detect the DAPI and A488 signals, respectively. The TL distribution and median TL were calculated with Life Length's proprietary program.

The length of each individual telomere is calculated by interpolation of the corresponding intensity of fluorescence into the regression curve prepared with the controls. A distribution of TL is thereafter calculated and the 20th percentile of said distribution is given in representation of the percentage of short telomeres. To remove machine variances over time, all samples (baseline, 3, 6, 9, and 12 months) were tested at the same time. Life Length was blinded during the analysis.

### Clinical laboratory assays

During visits at baseline and at the end of visits at 3, 6, 9, and 12 months after initiation of the test products (placebo or TA-65), vitals were checked and blood was drawn from each subject. Assays for a comprehensive metabolic panel (insulin, glucose, blood urea nitrogen, creatinine, estimated glomerular filtration rate, sodium, potassium, phosphorus, bilirubin, alkaline phosphatase, aspartate aminotransferase, and alanine aminotransferase), hematology panel (RBC, hemoglobin, hematocrit, complete blood count, white blood cells count, differential leukocytes, and platelets), lipid panel (total cholesterol, HDL cholesterol, triglycerides, and LDL cholesterol), inflammatory markers (C reactive protein and homocysteine), and immune cells including immunosenescence biomarkers (B lymphocytes, T lymphocytes, and Natural Killer cells) were carried out at Labco.

### Statistical analysis: multilevel model

Since each person was measured multiple times, the errors from a regression model would not be independent, thus violating one of the key assumptions of the model. To deal with this, we used a multilevel model. Because we were interested in nonlinear and possibly nonmonotonic relationships between time and median TL, we used month as a categorical variable. Alternatives such as spline models were considered and rejected because the number of time points per subject was relatively few. We used an unstructured covariance matrix based on fit indexes (Akaike information criterion). We included time, group, and their interaction in the model. The interaction term is most important, since it indicates whether the effect of time on median TL was different in the different groups.

## Results

### Median TL: baseline characteristics

**FIG. 1.**
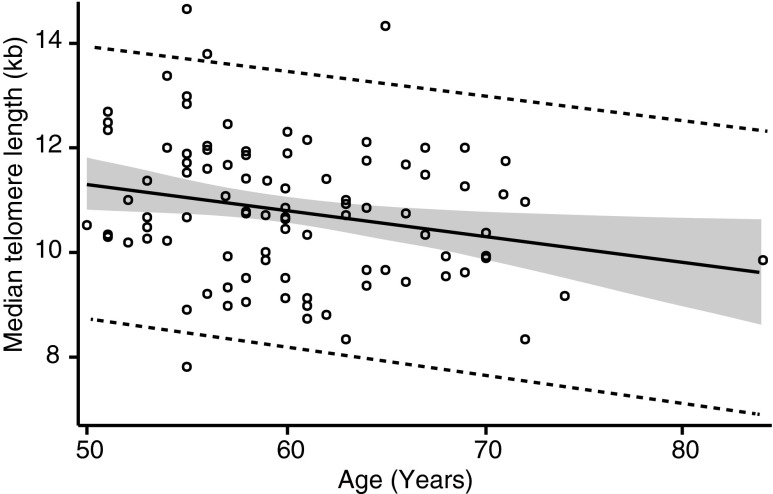
Baseline median TL. Least squares fit method is used to generate the linear regression model. Each bubble indicates a subject. Solid line indicates 95% confidence limits and broken line indicates 95% prediction limits. R^2^ is 0.056. The cross-sectional rate of change by age is −50 ± 21 (SE) bp/year. SE, standard error; TL, telomere length.

We used a linear regression model to analyze cross-sectional data of TLs of all 97 subjects at baseline. TL at baseline ranged from 7 to 15 kilo base pairs (kb) for the subjects aged from 53 to 87 years and was inversely correlated with age (R^2^ = 0.056). The cross-sectional rate of decline in TL for the baseline population was 50 ± 21 bp/year. Figure 1 shows the distribution of TL of the study participants at baseline.

### Average change in the median TL for TA-65 group and placebo group

Median TL was measured in the placebo group, low-dose TA-65 (250 U) group, and high-dose TA-65 (1000 U) group at baseline, 3, 6, 9, and 12 months (Table 1). At baseline, there were no significant differences in TL among the three groups, although the range of lengths was bigger for the placebo group. The TLs shown in Figure 1 are significantly longer than those measured in similar age-range cohorts by qPCR.^14^ The reason for this is likely due to the fact that FISH assays often detect signal from noncanonical telomeres (degenerate telomere sequences found in the sub-telomeric region at chromosome ends). It is also possible that telomere clustering in the hTP-qFISH over estimates TL, or that the methodology for assessing average TL is based on TRFs that may contain relatively large subtelomeric DNA.

**Table 1.** Average of Median TLs at Five Visits

	*Average of median TL (SD) in kb*				
*Group*	*Baseline*	*3 months*	*6 months*	*9 months*	*12 months*
Placebo	11.03 (1.49)	11.00 (1.38)	11.19 (1.28)	10.85 (1.36)	10.74 (1.55)
TA-65 (250 U)	10.57 (1.12)	10.92 (1.30)	10.89 (1.30)	10.92 (1.23)	10.81 (1.40)
TA-65 (1000 U)	10.44 (1.04)	10.86 (1.40)	10.59 (1.32)	10.61 (1.11)	10.22 (1.19)

The average TL in placebo, low-dose TA-65 (250 U), and high-dose TA-65 (1000 U) groups at baseline and at the end of 3, 6, 9, and 12 months in kilo base pairs (kb) with SD.

SD, standard deviation; TL, telomere length.

### Change in the median TL for TA-65 group versus placebo group: multilevel analysis

As discussed in the statistical methodology, to understand the nonmonotonic relationships with time and TL, a multilevel analysis was run. The effect of greatest interest was the interaction effect between time and group. The main effect of time tests whether the placebo changed over time, whereas the main effect of group tests whether the groups were different at baseline. Although both must be accounted for, our interest is in whether the three groups behaved differently over time and this is tested by the interaction (group and time interaction). It is important to distinguish between the raw data (shown in Table 1) and the parameter estimates by the multilevel analysis (shown in Table 2).

**Table 2.** Multilevel Model Analysis of Median TL Changes Compared with TL at Baseline

*Effect*	*Group*	*Time (months)*	*Change in TL (kb)*	*SE*	p
Group effect	Placebo	At baseline	Reference group		
	TA-65 (250 U)		−0.47	0.32	0.15
	TA-65 (1000 U)		−0.24	0.33	0.47
Time effect	Placebo	0	Reference group		
		3	−0.02	0.11	0.82
		6	0.16	0.09	0.07
		9	−0.17	0.09	0.07
		12	−0.29	0.10	0.01
Group and time effect	TA-65 (250 U)	0	Reference group		
		3	0.38	0.19	0.05
		6	0.16	0.16	0.34
		9	0.53	0.17	0.002
		12	0.53	0.18	0.005
	TA-65 (1000 U)	0	Reference group		
		3	0.25	0.20	0.22
		6	−0.13	0.17	0.46
		9	0.22	0.17	0.20
		12	−0.06	0.19	0.77

Placebo, low-dose TA-65 (250 U), and high-dose TA-65 (1000 U) groups are compared at baseline (0 months) and at the end of 3, 6, 9, and 12 months for median TL. The data show change in TL in comparison with that of the reference group(s). Results were adjusted for age and sex.

SE, standard error.

**FIG. 2.**
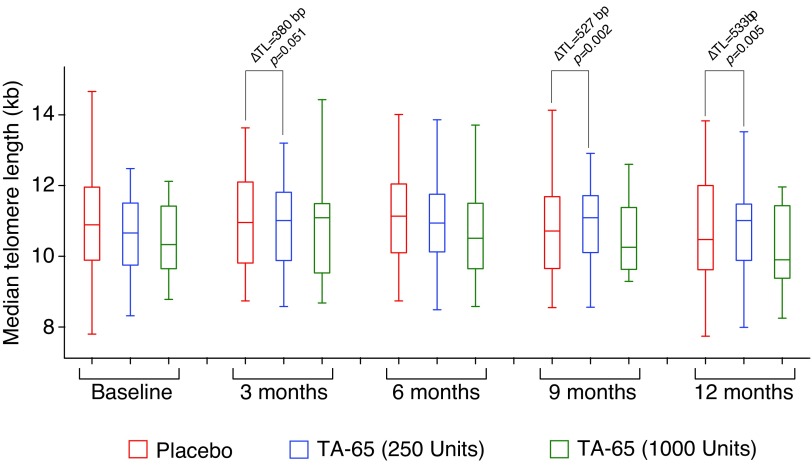
Change in the median TL compared with TL at baseline in placebo group, low-dose TA-65 (250 U) group, and high-dose TA-65 (1000 U) group. ΔTL represents change in TL compared with TL at baseline.

The TLs at baseline among the three groups were not significantly different as estimated by the group effect (Table 2). In the placebo group, there was a decrease in median TL at 9 and 12 months compared with that at baseline (Table 2 and Fig. 2). At 9 months the decrease was 170 ± 90 bp (*p* = 0.07) and at 12 months the decrease was 290 ± 100 bp (*p* = 0.01). Overall, the placebo group telomere data behaved slightly worse than expected (50–150 bp/year), which may be due to the CMV-positive status of the individuals tested. Also it may suggest that this cohort was either not as healthy at baseline as expected or perhaps had a relatively poor set of lifestyle behaviors.^15^

In the low-dose TA-65 (250 U) group, there was an increase in median TL at 3 months followed by relative stability (Table 2). Compared with that in the placebo group, the effect of time was significantly different in the TA-65 groups. The effect of low-dose TA-65 (250 U) on median TL was significantly higher at 9 months (median TL was 530 ± 170 bp longer, *p* = 0.002) and 12 months (again, median TL was 530 ± 180 bp longer, *p* = 0.005), and borderline was significantly higher at 3 months (median TL was 380 ± 190 bp longer, *p* = 0.05) but not significant at 6 months (Table 2 and Fig. 2).

The high-dose TA-65 (1000 U) showed a trend of improvement in TL compared with that in the placebo group, but the improvements did not reach statistical significance. It is not known why in this study the high-dose TA-65 (1000 U) group appeared to change in a random manner. This may have resulted from a compliance issue with subjects who took the higher dose. In future studies, it may be necessary to more tightly monitor compliance over time and increase the number of subjects and doses tested.

### Change in TL of the short telomeres (20th percentile) for TA-65 group and placebo group

**FIG. 3.**
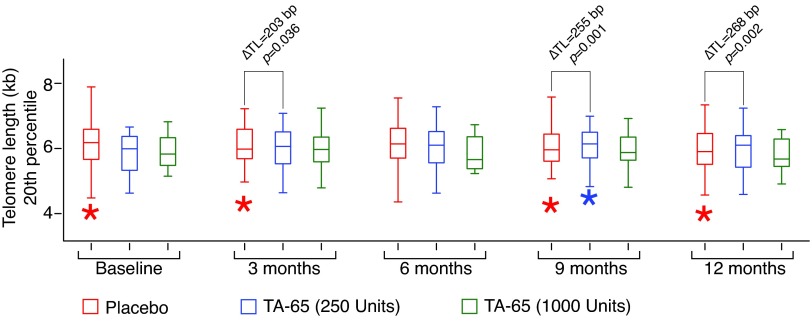
Change in the 20th percentile TL compared with TL at baseline in placebo group, low-dose TA-65 (250 U) group, and high-dose TA-65 (1000 U) group. ΔTL represents change in TL compared with TL at baseline. *Indicates outliers.

The shortest quintile of TL (<20th percentile) was measured in the placebo group, low-dose TA-65 (250 U) group, and high-dose TA-65 (1000 U) group at baseline, and at the end of 3, 6, 9, and 12 months (Fig. 3); the average lengths are represented in Table 3.

**Table 3.** Average of 20th Percentile TLs

	*Average of 20th percentile TL (SD) in kb*				
*Group*	*Baseline*	*3 months*	*6 months*	*9 months*	*12 months*
Placebo	6.14 (0.71)	6.08 (0.64)	6.17 (0.62)	6.01 (0.66)	5.97 (0.68)
TA-65 (250 U)	5.84 (0.68)	5.98 (0.78)	5.98 (0.81)	5.96 (0.72)	5.93 (0.49)
TA-65 (1000 U)	5.93 (0.49)	6.01 (0.64)	5.83 (0.52)	5.92 (0.52)	5.77 (0.52)

The average 20th percentile TL in placebo, low-dose TA-65 (250 U), and high-dose TA-65 (1000 U) groups at baseline and at the end of 3, 6, 9, and 12 months in kb with SD.

In the placebo group, there was a gradual decrease in average shortest quintile TL with time, as expected (Table 3). However, TL of the 20th percentile in the low-dose TA-65 (250 U) group increased at 3 months and was then relatively stable. In the high-dose TA-65 (1000 U) group, there was no consistent change in the 20th percentile TL (Table 3).

Again, the key results are whether the effect of time was different in the different groups. We found trends similar to those for the median. Here, the differences between the low-dose TA-65 (250 U) group and the placebo were significant at 3, 9, and 12 months (increment of 200, 260, and 270 bp, and the *p* values are 0.04, 0.001, and 0.002, respectively). Also as with the median length, the effects in the high-dose TA-65 (1000 U) group were inconsistent and nonsignificant (Table 4).

**Table 4.** Multilevel Model Analysis of Short TL Changes Compared with TL at Baseline

*Effect*	*Group*	*Time(months)*	*Change in TL (kb)*	*SE*	p
Group effect	Placebo	Baseline	Reference group		
	TA-65 (250 U)		−0.30	0.17	0.07
	TA-65 (1000 U)		−0.20	0.17	0.25
Time effect	Placebo	0	Reference group		
		3	−0.06	0.05	0.22
		6	0.03	0.05	0.54
		9	−0.13	0.04	0.0026
		12	−0.17	0.05	0.0005
Group and time effect	TA-65 (250 U)	0	Reference group		
		3	0.20	0.10	0.0358
		6	0.11	0.08	0.1763
		9	0.26	0.07	0.0009
		12	0.27	0.09	0.0023
	TA-65 (1000 U)	0	Reference group		
		3	0.10	0.10	0.30
		6	−0.14	0.09	0.11
		9	0.10	0.08	0.21
		12	−0.01	0.09	0.93

Placebo, low-dose TA-65 (250 U), and high-dose TA-65 (1000 U) groups are compared at baseline (0 months) and at the end of 3, 6, 9, and 12 months for 20th percentile TL. The estimate represents the change in TL in comparison with that in the reference group(s). Results were adjusted for age and sex.

### Key changes in the safety markers

Statistically significant differences between the baseline and 12 months measurements in the safety markers are shown in Supplementary Table S1 (Supplementary Data are available online at www.liebertpub.com/rej). There were no clinically significant changes in the safety markers during the study as judged by the physician (J.M.R.). Immune cell biomarkers were unfortunately inappropriately run and hence could not be used.

## Discussion

In a previous observational study, subjects taking TA-65 along with other supplements showed improvements from baseline in health biomarkers, especially in CMV^+^ subjects.^1^ Since the subjects were blind to their CMV status while taking TA-65, it is unlikely that the positive effects of TA-65 were due to a placebo effect. To confirm that there was in fact no significant placebo effect, this study was designed to be randomized, double blind, and placebo controlled. We tested a cohort of CMV^+^ subjects for the effect of TA-65 on TL. The TLs were measured using HT Q-FISH with automation to handle a large number of human samples and to improve consistency. The cross-sectional analysis of TL at baseline indicates a decline of 50 ± 21 bp/year, which is higher than in some studies, but consistent with other published data.^5,16,17^ The rate of telomere loss has been reported to be exacerbated in CMV^+^ individuals,^18^ which may also contribute to the relatively high rate of change in the cross-sectional analysis. The rate of loss reported in this study^18^ was 94 ± 9 bp/year in CMV^+^ subjects and 77 ± 9 bp/year in CMV^−^ subjects.

In this study, the placebo group had an average telomere attrition of 290 ± 100 bp/year (*p* = 0.01), whereas the low-dose TA-65 (250 U) group had net increase of 530 ± 180 bp/year (*p* = 0.005). Interestingly there were no statistically significant changes in TL in the high-dose TA-65 (1000 U) group. Loss of 290 bp/year in the placebo group is indeed large, but a large loss is to be expected in a group that is 100% CMV^+^ and consists of older individuals aged >60 years. The accelerated attrition is supported by: (1) CMV infection that causes significant shortening of TL in the age group of >60 years^18^ and (2) CMV seropositivity increases the oligoclonal expansion of the immune cells with age.^19^ Although variation in the rate of TL loss over time cannot be ruled out, there are limited studies on TLs in CMV subjects.

In the previous observational study,^1^ the subjects who took a very low starting dose of 5–10 mg/day of unformulated TA-65 (*i.e.*, active ingredient alone) had no significant change in TL. In this study, with an improvement in formulation (TA-65MD) to enhance bioavailability, the TA-65 250 U (with 8 mg of active ingredient) increased TL, whereas TA-65 1000 U (with 32 mg of active ingredient) showed no consistent changes in the TL. These data raise a possibility that TA-65 may have a bell-shaped dose response curve. Murine cell data suggest that TA-65 results in reduction of cells with short telomeres.^3^ It is possible that the high-dose TA-65 (1000 U), by increasing the short telomeres lengths, rescued the near senescent cells, resulting in a reduction in the median TL. A future study has been planned to address the expansion of near-senescent cells with additional TA-65 doses.

Analysis of the 20th percentile group showed trends similar to that of the overall group: TL increased in the low-dose TA-65 (250 U) group at 12 months (268 ± 85 bp), but there was no consistent change in the high-dose TA-65 (1000 U) group. The cause of no significant change over time in the high-dose TA-65 group is unknown and unexpected. However, there was a trend in the observational studies^1,2^ that high doses partially reverse some of the positive effects of TA-65.

Overall the most significant finding of this study was that the low-dose TA-65 (250 U) increased both median and short TLs in a statistically significant manner, which could have clinical significance as well. For example, TL has been positively associated with increased regenerative capacity of cells,^20,21^ reduced mortality and disease risks in humans,^22–24^ and increased resistance to infection.^25^

Based on the animal studies, the no-observed-adverse-effect level for oral TA-65 was considered to be greater than 150 mg/kg/bw/day in male and female rats, equivalent to 10,500 mg/day in a 70-kg individual, which is orders of magnitude higher than the highest doses seen in human pharmacokinetic studies. Note also that TA-65 is designated as GRAS^4^ and has been extensively tested for safety in humans. In addition, no cytotoxicity was identified in *in vitro* testing of 1 μM active ingredient on CD8^+^ T cells.^20^

The bulk of the evidence suggests that TA-65 lengthens telomeres by increasing telomerase activity. However, the dose response of TA-65 for TL could not be accurately ascertained with only two doses tested, and with one of the doses showing no significant change over time. For these reasons, a more highly powered study with three or more doses is being planned. In addition, the results from this study are consistent with the previous observations regarding the lack of any toxicity associated with the intake of TA-65. We did not find any product-related toxicities, as assessed by the biochemical markers of liver, kidney, and metabolic functions.

## Supplementary Material

Supplemental data
