# Supplementary material for: A Natural Product Telomerase Activator Lengthens Telomeres in Humans: A Randomized, Double Blind, and Placebo Controlled Study
Source: Rejuvenation Res. 2016 Dec 1;19(6):478–84. doi: 10.1089/rej.2015.1793 (PMC5178008; doi:10.1089/rej.2015.1793)
Supplement: Supplemental data [file Supp_Table1.pdf]

# Supplementary Data

Supplementary Table S1. The Measurements of Clinical Biomarkers

| Clinical parameters (reference range) | *Placebo* | | | *TA-65 (250 U)* | | | *TA-65 (1000 U)* | | |
|---|---|---|---|---|---|---|---|---|---|
| | *Baseline (SD)* | *12 months (SD)* | p | *Baseline (SD)* | *12 months (SD)* | p | *Baseline (SD)* | *12 months (SD)* | p |
| Gamma glutamyl transferase (<40 U/L) | 29.79 (22.32) | 31.69 (25.09) | 0.52 | 24.21 (13.96) | 25.22 (12.01) | 0.88 | 32.07 (20.37) | 28.82 (13.96) | 0.48 |
| Alkaline phosphatase (30–120 U/L) | **68.84 (18.53)** | **75.54 (22.96)** | **0.002** | 74.03 (17.52) | 74.47 (14.72) | 0.92 | 73.52 (15.94) | 75.45 (14.98) | 0.11 |
| Sodium (135–152 mmol/L) | 141.05 (2.71) | 141.00 (1.94) | 0.77 | 140.29 (2.94) | 140.26 (2.05) | 0.92 | **141.81 (2.82)** | **140.45 (2.42)** | **0.03** |
| Potassium (3.5–5.2 mmol/L) | **4.11 (0.32)** | **4.21 (0.29)** | **0.01** | 4.17 (0.34) | 4.18 (0.39) | 0.92 | 4.31 (0.42) | 4.32 (0.32) | 0.68 |
| Total bilirubin (0.3–1.2 mg/dL) | 0.62 (0.27) | 0.64 (0.31) | 0.63 | 0.58 (0.22) | 0.62 (0.17) | 0.30 | 0.60 (0.24) | 0.65 (0.24) | 0.59 |
| Total cholesterol (<200 mg/dL) | 219.04 (34.98) | 209.95 (31.66) | 0.15 | 216.74 (41.57) | 224.83 (45.14) | 0.98 | 228.48 (45.27) | 221.36 (43.42) | 0.30 |
| HDL cholesterol (male: >40 mg/dL female: >50 mg/dL) | 68.11 (15.73) | 67.16 (14.38) | 0.72 | 68.19 (9.85) | 70.78 (12.39) | 0.46 | 69.39 (17.50) | 69.86 (16.87) | 0.69 |
| LDL cholesterol (<100 mg/dL) | 128.98 (28.42) | 119.71 (26.94) | 0.05 | 129.63 (37.69) | 134.28 (45.16) | 0.89 | 136.13 (37.43) | 130.00 (40.03) | 0.41 |
| Triglycerides (<150 mg/dL) | 109.87 (52.51) | 114.97 (62.84) | 0.30 | 94.52 (48.61) | 99.17 (43.09) | 0.91 | 115.13 (38.71) | 107.07 (39.97) | 0.47 |
| C reactive protein (<6 mg/L) | 1.61 (1.58) | 2.42 (3.64) | 0.11 | 2.37 (2.51) | 2.30 (2.41) | 0.57 | 2.21 (2.21) | 2.58 (2.19) | 0.88 |
| Insulin (5–25 $\mu$5–25 n | 11.01 (3.89) | 11.63 (4.65) | 0.39 | 9.67 (2.87) | 8.91 (2.27) | 0.29 | 11.14 (3.91) | 10.32 (3.42) | 0.25 |
| Homocysteine (5–15 $\mu$mol/L) | 10.29 (3.21) | 10.31 (3.13) | 0.97 | 10.41 (3.66) | 11.12 (4.05) | 0.69 | 11.28 (3.57) | 10.97 (2.32) | 0.42 |
| Glucose (65–110 mg/dL) | 92.86 (9.41) | 93.79 (12.93) | 0.88 | 91.14 (6.71) | 90.17 (3.93) | 0.16 | 95.78 (12.69) | 96.59 (16.40) | 0.54 |
| Urea nitrogen (0–50 mg/dL) | 36.18 (8.42) | 34.10 (8.78) | 0.11 | 36.21 (6.38) | 37.83 (9.34) | 0.51 | 38.44 (9.09) | 38.82 (8.48) | 0.88 |
| Creatinine (<1.3 mg/dL) | **0.95 (0.14)** | **0.92 (0.14)** | **<0.001** | **0.93 (0.15)** | **0.91 (0.15)** | **0.03** | **0.98 (0.16)** | **0.95 (0.16)** | **0.02** |
| Uric acid (2–6.5 mg/dL) | 4.70 (1.25) | 4.96 (1.27) | 0.21 | 4.66 (1.18) | 5.22 (1.59) | 0.06 | **4.84 (1.29)** | **5.58 (1.41)** | **<0.001** |
| Alanine aminotransferase (<40 U/L) | 21.12 (8.19) | 21.46 (10.41) | 0.79 | 19.18 (8.33) | 18.70 (8.04) | 0.92 | 29.41 (35.14) | 27.50 (23.40) | 0.39 |
| Aspartate aminotransferase (<40 U/L) | 22.66 (5.33) | 22.56 (5.12) | 0.94 | 21.43 (5.11) | 21.39 (3.76) | 0.95 | 25.26 (11.35) | 25.18 (8.10) | 0.97 |
| Systolic BP (<120 mm Hg) | 127.54 (18.54) | 127.67 (17.47) | 0.55 | 127.61 (12.94) | 125.56 (14.93) | 0.21 | 132.56 (19.46) | 128.24 (10.02) | 0.26 |
| Diastolic BP (<80 mm Hg) | **76.44 (10.11)** | **74.46 (9.80)** | **0.03** | **81.04 (8.23)** | **74.78 (8.92)** | **<0.001** | **79.15 (10.25)** | **74.00 (10.02)** | **0.02** |

The measurements of markers and blood pressure are represented as mean (SD) for the placebo group, TA-65 250 U group, and TA-65 1000 U group at baseline and after 12 months (The other time points are not represented here). Bold numbers indicate the significant changes at 12 months compared with baseline.

SD, standard deviation.
